# High risk of peri‐implant disease in periodontal Ehlers–Danlos Syndrome. A case series

**DOI:** 10.1111/clr.13373

**Published:** 2018-10-08

**Authors:** Alexander Rinner, Johannes Zschocke, Anna Schossig, Rebekka Gröbner, Heinrich Strobl, Ines Kapferer‐Seebacher

**Affiliations:** ^1^ Private Practice Stanz Austria; ^2^ Division of Human Genetics Medical University of Innsbruck Innsbruck Austria; ^3^ Clinical Department of Maxillofacial Surgery Innsbruck Medical University Innsbruck Austria; ^4^ Department of Operative and Restorative Dentistry Medical University of Innsbruck Innsbruck Austria

**Keywords:** Ehlers–Danlos, hypermobility, peri‐implantitis, periodontitis, rare disease

## Abstract

**Objectives:**

Periodontal Ehlers–Danlos syndrome (pEDS) has recently been delineated as a molecularly defined cause of early severe periodontitis. Here we report that implant treatment failed in three affected individuals from one family.

**Materials and Methods:**

Longitudinal data before and after implant treatment were examined for three individuals with genetically confirmed pEDS in the course of a large‐scale pedigree analysis.

**Results:**

Most detailed information was available for individual 1 in whom first periodontal bone loss was diagnosed at age 16 years. Rapid progression resulted in multiple tooth extractions at age 23 years and interforaminal placement of four implants. After primary implant success, peri‐implant bone loss accompanied by highly inflamed tissues and receding gums led to explantation five years later. In individual 2, severe periodontitis was diagnosed at age 15 years and resulted in extraction of all mandibular teeth at age 28 years. Four interforaminal implants were placed. Peri‐implant bone loss was diagnosed four years later, when up to three implant threads were exposed. Individual 3 showed complete tooth loss at age 29 years. He was restored with ten implants and removable prosthesis. Peri‐implant bone loss was diagnosed radiologically eight years later, when seven implant threads were exposed.

**Conclusion:**

This is the first report on severe peri‐implant bone loss in pEDS. Retention of teeth as long as possible is the primary objective in pEDS as satisfying prosthetic solutions are missing. Further evaluation of dental management in individuals with pEDS is needed to develop concise treatment guidelines.

## 1 INTRODUCTION

Ehlers–Danlos syndromes (EDS) are a clinically and genetically heterogeneous group of hereditary connective tissue disorders (Brady, et al., 2017; Malfait, et al., 2017). Periodontal EDS (pEDS, previously EDS type VIII) is a specific EDS subtype with autosomal‐dominant inheritance. It is caused by mutations in *C1R* and *C1S* genes, encoding the first component of the classical complement pathway (Kapferer‐Seebacher, et al., 2016). The clinical features of pEDS appear to be linked to both, structural alterations of the connective tissue as well as a disturbance of immunological functions. Major criteria for the clinical diagnosis of pEDS are:

1. early severe periodontitis (before age 30 years, usually in childhood)
2. a specific gingival phenotype characterized by the lack of attached gingiva,
3. pretibial hemosiderotic plaques, and
4. bruising out of proportion to trauma.

Additional clinical features include (mild) joint hypermobility, skin fragility with abnormal scarring, an increased rate of infections, aneurisms, organ ruptures, and hoarse voice (Kapferer‐Seebacher, et al., 2016; Kapferer‐Seebacher, Lundberg, Malfait, & Zschocke, 2017; Malfait, et al., 2017).

Up to now, 30 case reports and pedigree analyses including 130 individuals with pEDS have been reported (Kapferer‐Seebacher, et al., 2017). Little is known about the effectiveness of different dental treatment strategies. Here we provide the first report on three affected individuals, indicating that implant therapy has a high risk for failure in pEDS.

## 2 MATERIAL AND METHODS

### 2.1 Subjects

In 2016, a large‐scale pedigree analysis identified 93 individuals clinically and genetically diagnosed with pEDS (Kapferer‐Seebacher, et al., 2016). The present study cohort includes three members of the Austrian five‐generation family treated with dental implants (Kapferer‐Seebacher, et al., 2016): 1‐IV‐2 (individual 1), 1‐III‐2 (individual 2), and 1‐III‐3 (individual 3). No further individuals with dental implants were available for clinical investigation.

Clinical features suggestive for pEDS were early severe periodontitis, lack of attached gingiva, mild joint hypermobility of the digits and the elbows, easy bruising, and velvety skin. There were no pretibial discolorations. Sjögren´s syndrome was diagnosed in individuals 1 and 2 at age 33 and 35 years (y), respectively. Scoliosis was diagnosed in individual 1 at age 22 y. There were no other clinical findings previously reported with pEDS (see Supporting information Data S1 (Kapferer‐Seebacher, et al., 2016)). Exome sequencing identified a heterozygous *C1R* missense mutation (c.149_150TC>AT, p.Val50Asp) which was also present in all other affected family members (Kapferer‐Seebacher, et al., 2016).

### 2.2 Dental examinations

The diagnosis of early severe periodontitis was based on radiologic evidence of severe alveolar bone loss (≥50%) at an age of ≤25 years, or history of complete tooth loss at an age of ≤30 years. The diagnosis of peri‐implantitis was based on the radiographic evidence of exposure of ≥1 implant thread. Dental x‐rays and details on dental history were collected from former dentists.

### 2.3 Ethical considerations

The study was conducted in accordance with the Helsinki Declaration of 1975, as revised in 2013, and was approved as part of the Biobank for Rare diseases by the Ethics Committee of the Medical University Innsbruck, Austria (Study No. UN4501). The manuscript has been prepared in compliance with the appropriate EQUATOR guidelines (CARE).

### 2.4 Individual 1

#### 2.4.1 Dental history

**Figure 1:**
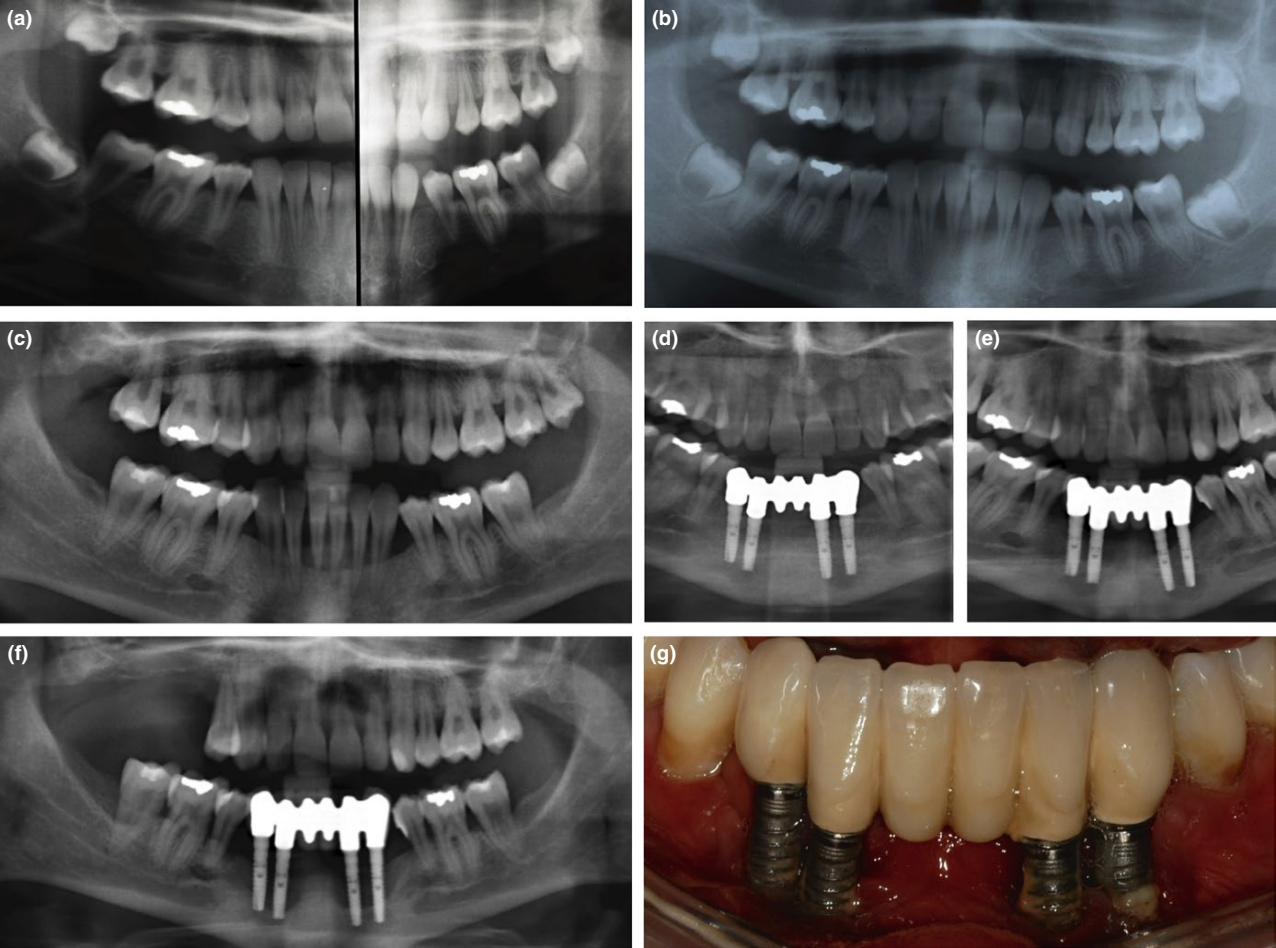
Periodontal and peri‐implant bone loss in individual 1. (a) Splitted orthopantomogram at age 14 years: At the end of orthodontic treatment, no periodontal bone loss is evident. (b) Orthopantomogram at age 16 years and (c) age 18 years: Periodontal bone loss with rapid progression is clearly visible in the mandibular anterior region. (d) Implant placement after bone augmentation, and prosthetic reconstruction at age 24 years. (e) Peri‐implant bone loss 3 years, and (f) 5 years after implantation. (g) Rapid progressing peri‐implantitis was clinically characterized by the absence of pocketing, but receding gums and exposed implant threads

Individual 1 is a never‐smoking 33‐year‐old woman. The first available radiograph at an age of 7 y shows the patient in age‐appropriate mixed dentition. At age 9 y, orthodontic treatment was started (removable orthodontic appliances in the mandibula, and brackets in the maxillary front). No periodontal bone loss was evident during and after orthodontic treatment (Figure 1a). Oral hygiene instructions and biofilm management were regularly provided because of severe gingival inflammation. First periodontal bone loss was diagnosed radiologically at age 16 y, when approximately 50% of bone had been lost in the mandibular first incisors (Figure 1b). Rapid progression of bone loss despite regular periodontal debridement and oral hygiene instructions was documented by the preceding dentist with dental x‐rays until the age 23 y, when the lower anterior teeth 33 to 43 were extracted (Figure 1c). No formal oral hygiene indices were recorded but in the patient file it was stated several times that oral hygiene was appropriate. The patient used an electric toothbrush (Sonicare, Philips Oral Healthcare, Snoqualmie, USA), interdental brushes (Curaprox, Curaden International AG, Kriens, Switzerland) and dental floss.

#### 2.4.2 Oral rehabilitation

The patient presented at age 24 y at the private practice of A.R. The first tentative diagnosis was localized aggressive periodontitis. Mean clinical attachment loss (CAL) was 3.42 ± 1.96 mm, 21 teeth had a CAL >5 mm. Mean probing pocket depth was 2.87 ± 0.97 mm (range 1–4 mm), with 30% of sites measuring 4 mm of pocket depth and no pocket depths ≥5 mm. The plaque control record measured at six sites per tooth (PCR)(O'Leary, Drake, & Naylor, 1972) was 36%, bleeding on probing (BoP) was 36%. Microbiological samples were taken from four subgingival sites,pooled, and analyzed by PCR‐analysis for the presence of 11 periodontopathogenic bacteria with the micro‐IDent® plus test (Hain Lifescience, D‐72147 Nehren, Germany). *Tannerella forsythia*,* Treponema denticola*, and *Prevotella intermedia* were detected at low concentrations; below detection levels were *Aggregatibacter actinomycetemcomitans, Porphyromonas gingivalis, Parvimonas micra, Fusobacterium nucleatum, Campylobacter rectus, Eikenella corrodens, Eubacterium nodatum,* and *Campylobacter species*.

Periodontal therapy consisted of oral hygiene instructions and periodontal debridement. The mandibular height was reconstructed with a cortico‐cancellous bone block taken from the anterior iliac crest. Four implants (alphatech® TL, 3.4x14mm, Henry Schein Dental, Langen, Germany) were inserted under general anesthesia in locations 33, 32, 42, and 43 by the same surgeon who had performed the grafting procedure. Implants were uncovered eight months after placement. Stainless steel solid abutments (alphatech®, Henry Schein Dental, Langen, Germany) and a porcelain‐fused‐to‐metal (alloy of gold Herador H, Heraeus) cemented bridge were selected as prosthetic reconstruction (Figure 1d). No clinical or radiologic signs of implant‐related inflammation were observed at that time.

#### 2.4.3 Follow‐up

Twelve months later, peri‐implant bone loss was radiologically apparent; two years later two to four implant threads were radiologically and intraorally exposed in all implants. There was no pocketing, but receding gums with highly inflamed peri‐implant soft tissues. Peri‐implant probing depths were 2.89 ± 0.98 mm (range 1–4 mm) and mucosal recession was 3.75 ± 1.13 mm (range 1–5 mm). Monthly debridement with sonic scalers and curettes and oral hygiene instructions could not stop the rapid progressing peri‐implant bone loss, resulting in multiple extractions and explantation 5 y after implantation, at age 29 y (Figure 1e–f). The patient received metal frame partial dentures. In 2014, at age 30 y, the accurate clinical diagnosis of pEDS was made at the Medical University of Innsbruck.

### 2.5 Individual 2

#### 2.5.1 Dental history

**Figure 2:**
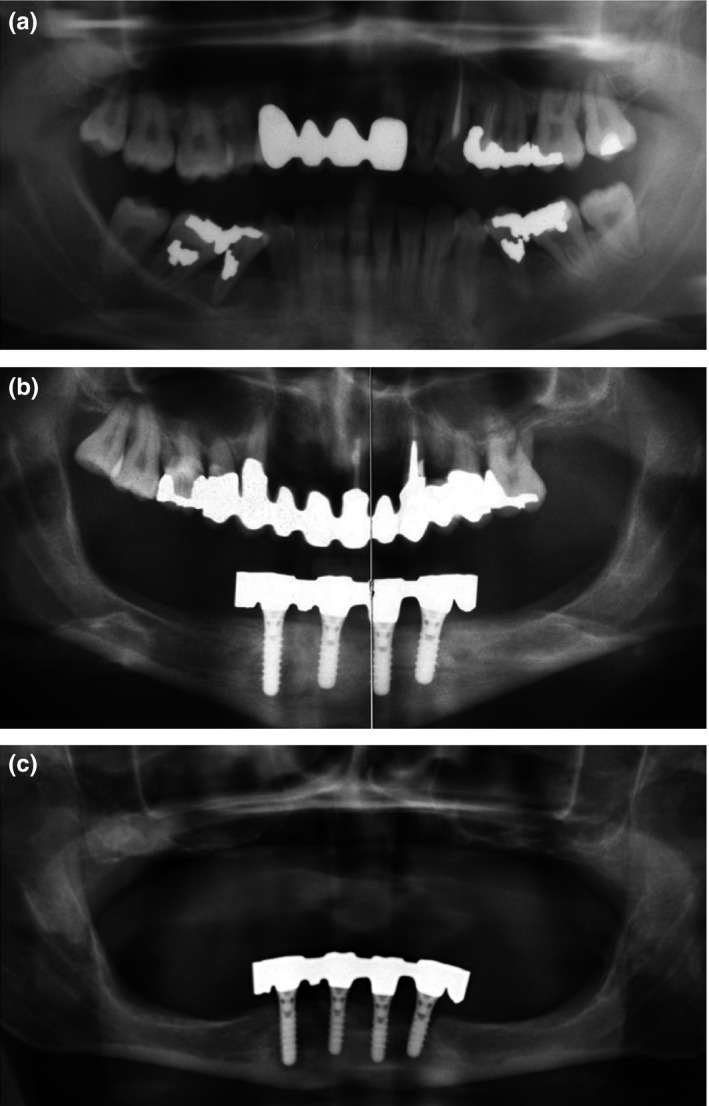
Periodontal and peri‐implant bone loss in individual 2. (a) Severe periodontal bone loss is evident in the lower jaw on the first available dental *x*‐ray at age 24 years. (b) Implant placement and prosthetic reconstruction at age 45 years. (c) Peri‐implant bone loss 7 years after implant placement. All maxillary teeth had been extracted due to periodontal disease

Individual 2 is the 56 y old mother of individual 1, a never‐smoker. She reported on first periodontal tooth loss at age 15 y. Dental x‐rays were available for the last 32 y. The first available radiograph at an age of 24 y shows severe periodontal bone loss. Rapid progression of bone loss despite regular periodontal debridement and oral hygiene instructions was documented by the preceding dentist with dental x‐rays until the age 28 y, when all mandibular teeth were extracted (Figure 2a). At age 45 y, four interforaminal titanium implants (Straumann, Basel, Switzerland) were inserted under local anesthesia. The mandibula was restored with an individually milled gold bar and removable full dentures (Figure 2b). Three years later, severe peri‐implant bone loss was radiologically evident as exposure of one to four implant threads (Figure 2c); all maxillary teeth had been extracted, and the patient had received a removable full denture in the maxilla.

The patient presented at age 49 y at the practice of A.R. At that time, peri‐implant probing depths were 3.33 ± 1.2 mm and mucosal recession was 3.71 ± 1.2 mm. PCR and BoP were 67%. Regular debridement with sonic scalers, curettes, and air‐powder polishing was performed but had no effect on peri‐implant bone loss and inflammation. Peri‐implant bone loss decelerated after menopause approximately at age 54 y.

### 2.6 Individual 3

**Figure 3:**
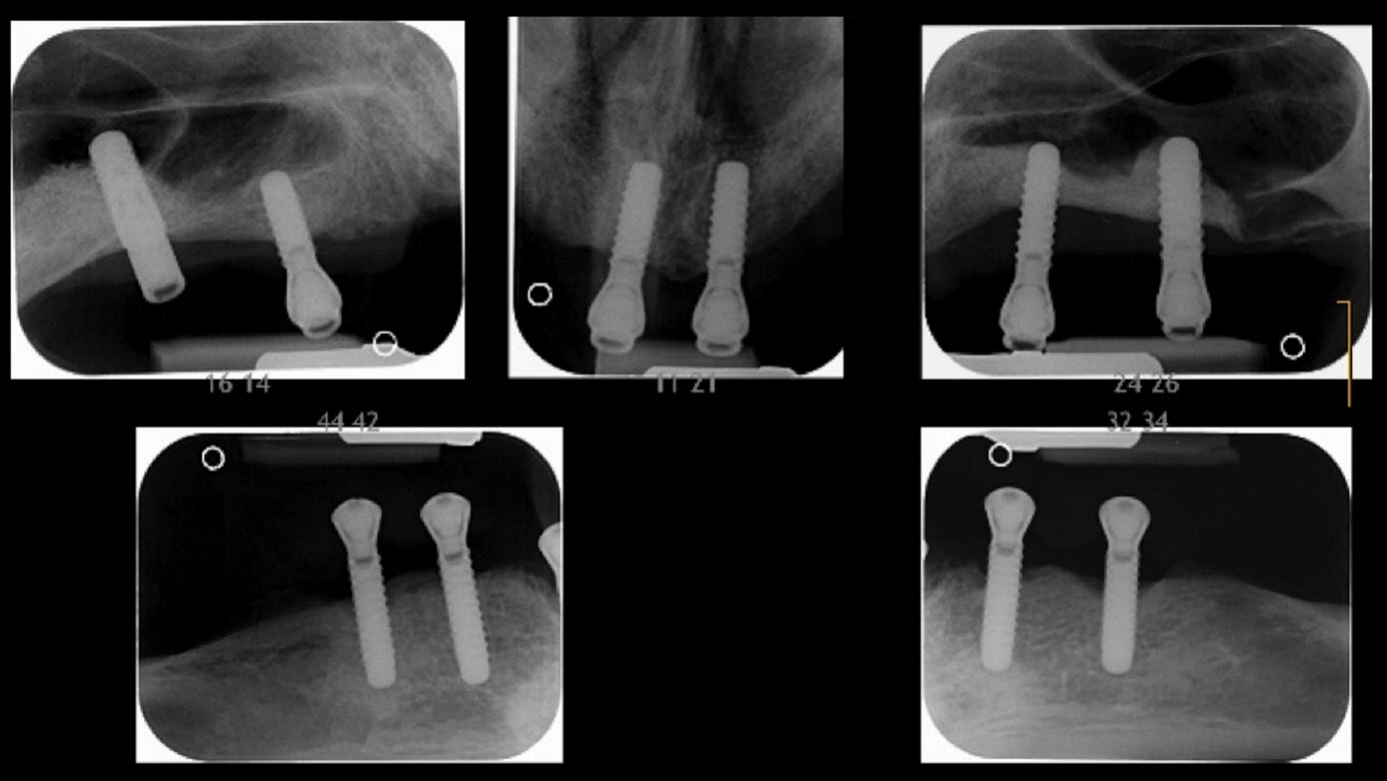
Periodontal and peri‐implant bone loss in individual 3. Severe peri‐implant bone loss was diagnosed radiologically 8 years after implant placement

The brother of individual 2 reported complete tooth loss due to periodontal disease at age 29 y. At age 40 y, he received six maxillary and four mandibular implants. Severe peri‐implant bone loss was diagnosed radiologically at age 48 y when up to seven implant threads were exposed (Figure 3). At that time, PCR was 29% and BoP was 36%. Mean peri‐implant probing depths were 3.37 ± 0.55 mm and mucosal recession was 2.87 ± 1.18 mm (range 1–6 mm). The individual was not available for further investigations or follow‐up analysis.

## 3 DISCUSSION

Periodontal EDS is a rare disease which can be clinically diagnosed by dental professionals. Since the systemic manifestation may be subtle the dental evaluation can be crucial for the diagnosis of the condition. Defining clinical criterion is early severe periodontitis in association with lack of attached gingiva, receding gums, and pretibial discolorations. Pretibial discolorations are caused by frequent hematomas due to easy bruising in combination with abnormal scarring, which results in persisting hemosiderotic plaques. In the present individuals, pretibial discolorations were missing and connective tissue features were mild or even missing, which confused the clinical diagnosis. Organ ruptures, hernias, scoliosis at an early age and joint hypermobility were reported in other family members (Kapferer‐Seebacher, et al., 2016).

The suspected clinical diagnosis of pEDS should be validated by genetic testing of the *C1R* and *C1S* genes. Complement 1 subcomponents r and s (C1r and C1s) are serine proteases which play a role in the activation of the classical complement cascade, a major element of antimicrobial host defense through its ability to recognize pathogens and limit infection in the early phase after exposure to microorganisms. The pathogenic link between mutations in complement 1 component genes and connective tissue alterations is currently under investigation. One potential explanation is abnormal interaction of mutant C1r and/or C1s with collagen proteins as binding of C1r‐C1s to the third subcomponent C1q, resulting in the active C1 complex, is mediated by a highly conserved collagenous domain (Venkatraman Girija, et al., 2013).

Both, structural alterations of the connective tissue as well as disturbances of the immunologic function appear to contribute to periodontal and peri‐implant disease in pEDS, although the exact pathomechanism is unknown. Structural alterations of oral connective tissues are evident as lack of attached gingiva and thin oral tissues. Hyperinflammation is already recognized in early childhood as severe gingivitis in the presence of even mild biofilm accumulation. Early severe periodontitis with rapid progression is usually diagnosed in childhood (mean age 12 years, range 2 to 29 years)(Kapferer‐Seebacher, et al., 2017).

In the present report, biofilm and bleeding indices were not measured by the former dentist, and the level and impact of oral biofilm accumulation on peri‐implant disease progression cannot be evaluated. We assume that similar to periodontal disease in pEDS, peri‐implant disease is characterized by severe inflammation and disease progression even in the presence of only mild biofilm accumulation. Strict biofilm control might stop the disease.

Peri‐implant bone loss in the present individuals was not accompanied by pocketing but by receding soft tissues and intraorally exposed implant threads. For titanium surfaces, it was proposed that they activate complement through the classical pathway and that C3 deposition on the surfaces enhances complement activation in a positive amplification loop via the alternative pathway (Walivaara, Askendal, Lundstrom, & Tengvall, 1996); however, this was never validated. Peri‐implant bone loss in individual 1 exacerbated during each pregnancy. High levels of estrogen during pregnancy are associated with reduced levels of C1 inhibitor (Derzsy, Prohaszka, Rigo, Fust, & Molvarec, 2010). In her mother, peri‐implant disease arrested with menopause, implicating that estrogen is a trigger for peri‐implant disease in pEDS.

There are many unresolved questions concerning dental treatment of individuals with pEDS. In single individuals, we observed rapid progression of attachment loss when teeth are loaded with prosthetic devices like clasps. Therefore, we hypothesized that periodontal breakdown in pEDS is strongly influenced by mechanical loading. However, this is not supported by the observation in individual 1 that orthodontic treatment did not result in periodontal bone loss. The present report on peri‐implant disease is limited by the low number of cases, a well‐known problem with rare diseases. International collaborations and databases are needed to increase case numbers.

In conclusion, peri‐implant bone loss has been diagnosed in all three pEDS individuals followed by us who received dental implants. Our observations indicate that the retention of teeth as long as possible is the primary goal as prosthetic solutions in pEDS are missing. Further reports on dental treatment and long‐term follow‐up of individuals diagnosed with pEDS are needed to develop concise treatment guidelines.

## **CONFLICT OF INTEREST**

The authors declare that there are no conflict of interests in this study.

## References

[clr13373-bib-0001] Brady, A. F. , Demirdas, S. , Fournel‐Gigleux, S. , Ghali, N. , Giunta, C. , Kapferer‐Seebacher, I. , … Malfait, F. (2017). The ehlers‐danlos syndromes, rare types. American Journal of Medical Genetics Part C, Seminars in Medical Genetics, 175, 70–115. 10.1002/ajmg.c.31550. 28306225

[clr13373-bib-0002] Derzsy, Z. , Prohaszka, Z. , Rigo, J. Jr , Fust, G. , & Molvarec, A. (2010). Activation of the complement system in normal pregnancy and preeclampsia. Molecular Immunology, 47, 1500–1506. 10.1016/j.molimm.2010.01.021. 20181396

[clr13373-bib-0003] Kapferer‐Seebacher, I. , Lundberg, P. , Malfait, F. , & Zschocke, J. (2017). Periodontal manifestations of ehlers‐danlos syndromes: A systematic review. Journal of Clinical Periodontology, 44, 1088–1100. 10.1111/jcpe.12807. 28836281

[clr13373-bib-0004] Kapferer‐Seebacher, I. , Pepin, M. , Werner, R. , Aitman, T. J. , Nordgren, A. , Stoiber, H. , ... Wilflingseder, D. (2016). Periodontal ehlers‐danlos syndrome is caused by mutations in c1r and c1s, which encode subcomponents c1r and c1s of complement. American Journal of Human Genetics, 99, 1005–1014. 10.1016/j.ajhg.2016.08.019. 27745832 PMC5097948

[clr13373-bib-0005] Malfait, F. , Francomano, C. , Byers, P. , Belmont, J. , Berglund, B. , Black, J. , … Tinkle, B. (2017). The 2017 international classification of the ehlers‐danlos syndromes. American Journal of Medical Genetics Part C, Seminars in Medical Genetics, 175, 8–26. 10.1002/ajmg.c.31552. 28306229

[clr13373-bib-0006] O'Leary, T. J. , Drake, R. B. , & Naylor, J. E. (1972). The plaque control record. Journal of Periodontology, 43, 38 10.1902/jop.1972.43.1.38. 4500182

[clr13373-bib-0007] Venkatraman Girija, U. , Gingras, A. R. , Marshall, J. E. , Panchal, R. , Sheikh, M. A. , Gal, P. , … Wallis, R. (2013). Structural basis of the C1q/C1s interaction and its central role in assembly of the C1 complex of complement activation. Proceedings of the National Academy of Sciences, 110(34), 13916–13920. 10.1073/pnas.1311113110 PMC3752233 23922389

[clr13373-bib-0008] Walivaara, B. , Askendal, A. , Lundstrom, I. , & Tengvall, P. (1996). Blood protein interactions with titanium surfaces. Journal of Biomaterials Science, Polymer Edition, 8, 41–48. 8933289 10.1163/156856297x00560

